# ABCA7 and Pathogenic Pathways of Alzheimer’s Disease

**DOI:** 10.3390/brainsci8020027

**Published:** 2018-02-05

**Authors:** Tomonori Aikawa, Marie-Louise Holm, Takahisa Kanekiyo

**Affiliations:** Department of Neuroscience, Mayo Clinic, 4500 San Pablo Road, Jacksonville, FL 32224, USA; aikawa.tomonori@mayo.edu (T.A.); holm.marie-louise@mayo.edu (M.-L.H.)

**Keywords:** ABCA1, amyloid-β, amyloid precursor protein, cholesterol, genetics, macrophage, microglia, neurons, phagocytosis, phospholipids

## Abstract

The ATP-binding cassette (ABC) reporter family functions to regulate the homeostasis of phospholipids and cholesterol in the central nervous system, as well as peripheral tissues. ABCA7 belongs to the A subfamily of ABC transporters, which shares 54% sequence identity with ABCA1. While ABCA7 is expressed in a variety of tissues/organs, including the brain, recent genome-wide association studies (GWAS) have identified ABCA7 gene variants as susceptibility loci for late-onset Alzheimer’s disease (AD). More important, subsequent genome sequencing analyses have revealed that premature termination codon mutations in ABCA7 are associated with the increased risk for AD. Alzheimer’s disease is a progressive neurodegenerative disease and the most common cause of dementia, where the accumulation and deposition of amyloid-β (Aβ) peptides cleaved from amyloid precursor protein (APP) in the brain trigger the pathogenic cascade of the disease. In consistence with human genetic studies, increasing evidence has demonstrated that ABCA7 deficiency exacerbates Aβ pathology using in vitro and in vivo models. While ABCA7 has been shown to mediate phagocytic activity in macrophages, ABCA7 is also involved in the microglial Aβ clearance pathway. Furthermore, ABCA7 deficiency results in accelerated Aβ production, likely by facilitating endocytosis and/or processing of APP. Taken together, current evidence suggests that ABCA7 loss-of-function contributes to AD-related phenotypes through multiple pathways. A better understanding of the function of ABCA7 beyond lipid metabolism in both physiological and pathological conditions becomes increasingly important to explore AD pathogenesis.

## 1. Introduction

Alzheimer’s disease (AD) is the leading cause of dementia in the elderly, accounting for 60–80% of cases. Approximately 5.5 million individuals are living with Alzheimer’s dementia in the United States. This number is estimated to increase continuously due to the expansion of the aged population [1]. Alzheimer’s disease is pathologically characterized by the presence of amyloid-bearing plaques and neurofibrillary tangles, which are often accompanied by neuronal loss and trigger innate immune responses in the brain [2,3,4]. While AD is a neurodegenerative disease with complex pathogenesis, several genetic factors have been associated with the development of the disease [5]. Although a small population size (<0.5%), dominantly inherent mutations in three genes encoding amyloid precursor protein (APP), presenilin 1 (PSEN1), and presenilin 2 (PSEN2), cause familial AD, usually at a young age (30–50 years of age) [6]. Each of these genes has been shown to accelerate the production of neurotoxic amyloid-β (Aβ), leading to the accumulation and deposition of Aβ in the brain. On the other hand, the majority of AD cases are sporadic and late-onset, occuring in individuals over the age of 65, sharing the same clinical and pathological features with familial type early-onset AD [7]. Importantly, several genomic variations are attributable to 60–80% of cases of late-onset AD [8]. While the ε4 allele of *APOE* is the strongest genetic risk factor for late-onset AD [9,10,11,12,13,14,15,16], several gene loci in *ABCA7* on chromosome 19p13.3 have also been recognized as novel risk factors for the disease [17]. *ABCA7* codes ATP-binding cassette (ABC) transporter A7, which is a member of the A subfamily of (ABC) transporters. Consistently, accumulating in vitro and in vivo studies support the potential contribution of ABCA7 to AD-related phenotypes. Therefore, to explore the pathogenesis of AD, a greater understanding of the role of ABCA7 in physiological and pathological conditions might be important. In this review, we summarize current evidence for the risk of *ABCA7* gene variants of AD development and discuss how ABCA7 is involved in the pathogenic pathways of AD.

## 2. *ABCA7* Gene Variants and Alzheimer’s Disease

Common variants of *ABCA7* with a minor allele frequency (MAF) of more than 5% have been implicated to associate with the risk for AD [17]. In 2011, Hollingworth et al. identified the common SNP (single nucleotide polymorphism) variant rs3764650, which is located in an *ABCA7* intron, as one of the susceptibility loci for late-onset AD (odds ratio [OR] = 1.23; 95% CI = 1.17–1.28) with replication among independent Caucasian cohorts through a genome-wide association study (GWAS) [18]. Naj et al. also reported that *ABCA7* SNP rs3752246, a missense variant (p.Gly1527Ala), is associated with the risk for late-onset AD (OR = 1.15; 95% CI = 1.09–1.21) [19]. Furthermore, a large meta-analysis of GWAS in individuals of European ancestry identified a new susceptibility variant rs4147929 in an *ABCA7* intron (OR = 1.15; 95% CI = 1.11–1.19) [20]. Interestingly, *ABCA7* rs3764650 has been associated with cortical and hippocampal atrophy in cognitively normal and mild cognitive impairment (MCI) subjects [21], as well as with memory decline in MCI and late-onset AD patients [22]. Therefore, *ABCA7* is possibly responsible for both the development and progression of AD.

In an African American cohort, a coding variant of *ABCA7* rs3764647 (p.His395Arg), located near rs3752246, has been associated with AD risk (OR = 1.32; 95% CI = 1.07–1.63), while no or a minimal significant association was detected in rs3752246 and rs3764650, respectively [23]. Another study in African Americans revealed that *ABCA7* rs115550680 is linked to the development of late-onset AD, in which the effect size (OR = 1.79; 95% CI = 1.47–2.12) is comparable with that of *APOE* ε4 (OR = 2.31; 95% CI = 2.19–2.42) [24]. In addition, although *ABCA7* rs142076058 (p.Arg578Alafs) is likely rare in Caucasians, it is relatively common in African Americans and has been identified as an AD risk allele; MAF 15.2% in AD vs. 9.74% in controls (OR = 2.13; 95% CI = 1.42–3.20) [25]. Thus, while increasing evidence clearly indicates that *ABCA7* gene variants are involved in AD risk in both Caucasians and African Americans, there may be ethnic-dependent effects.

In addition to the common variants, whole genome sequencing, exome sequencing, and targeted resequencing have also demonstrated that some of the low frequency variants (MAF 1–5%) and rare variants (MAF < 1%) in *ABCA7* have significant associations with the risk for AD. In a Belgian cohort, a low frequency variant, rs78117248, in an *ABCA7* intron showed a strong association with AD even after adjustment for the common SNPs, rs3764650, rs4147929, and rs3752246 (OR = 2.00, 95% CI 1.22–3.26) [26]. A rare *ABCA7* missense variant (rs3752239; p.Asn718Thr) was also shown to contribute to AD risk in African Americans [27]. On the other hand, another study showed that a low-frequency coding variant, rs72973581 (p.G215S), is a protective allele against AD (OR = 0.57; 95% CI = 0.41–0.80) in British and North-American ancestry, although the association is modest [28]. Of note, in 2015, Steinberg et al. comprehensively analyzed rare premature termination codon (PTC) mutations in *ABCA7* using whole genome sequencing and demonstrated that they are associated with AD risk in an Icelandic population; when analyzed by combining those rare “loss-of-function” variants, the OR is calculated to be 2.12 [29]. Several independent studies have also confirmed the association of *ABCA7* loss-of-function variants with increased AD risk [26,30,31,32,33,34,35]. Interestingly, long-read MinION cDNA sequencing has revealed that some of the *ABCA7* loss-of-function variants receive exon skipping or alternative splicing, which likely allows the production of functional proteins and rescues the deleterious effects [34]. Taken together, accumulating evidence through genetic studies suggests that the contribution of *ABCA7* to AD risk is mediated by the dysfunction or reduction of ABCA7. Indeed, a common *ABCA7* variant, rs3764650, likely influences ABCA7 expression levels in the brain. Whereas ABCA7 mRNA expression is increased in AD brains compared to control individuals, carrying the protective rs3764650 (T) allele is associated with higher ABCA7 expression levels [36].

## 3. Biochemical and Functional Features of ABCA7

### 3.1. ABCA7 Structure

**Figure 1:**
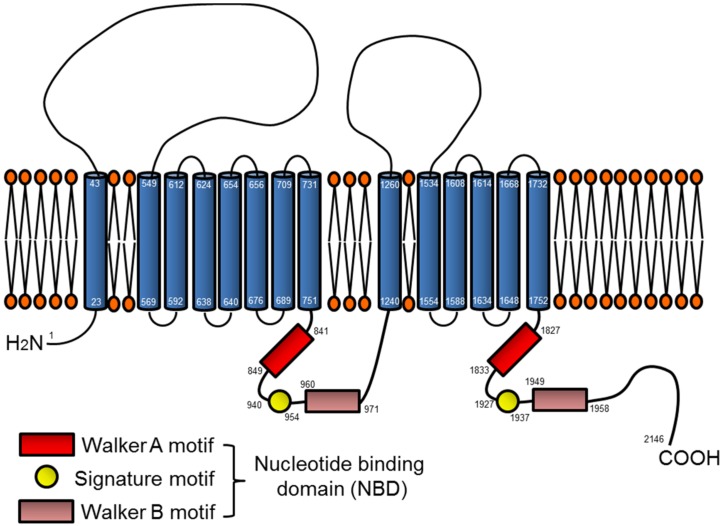
Topological model of ABCA7. The full-length ABCA7 is predicted to possess two hydrophobic transmembrane domains and two large loops serving as substrate-binding domains by OCTOPUS [39]. In addition, ABCA7 has two nucleotide binding domains (NBDs) composed of three motifs: Walker A, Walker B, and the signature motifs [40]. Lipid species are transported across the membrane bilayer through binding of ATP to the NBDs.

ATP-binding cassette (ABC) transporters constitute a superfamily of highly conserved proteins involved in the membrane transport of various substrates, such as ions, amino acids, lipids, and sterols across cell membranes [37]. Absolute ABC transporters are characterized by two nucleotide binding domains (NBD), which contain conserved Walker A and B motifs and conserved sequences, as well as two transmembrane domain bundles each composed of six membrane-spanning helices. The specificity of the transported molecules appears to be determined by the transmembrane domains, while ATP is required for the transport activity at the NBD [38]. ABCA7 possesses a typical ABC transporter structure, composed of 2146 amino acids with a molecular weight of approximately 220 kDa [39], which is mainly localized in the plasma membrane and the Golgi apparatus [40]. Of the 12 A class members, ABCA7 and ABCA1 are the closest homologues, sharing 54% sequence identity [39] (Figure 1). In vitro studies in HEK293 cells transfected with ABCA7 and ABCA1 have shown that ABCA7 also shares functional attributes with ABCA1; Apolipoprotein A (apoA)-I induces the release of cellular lipids including cholesterol and phospholipids through the two extracellular domains commonly preserved in ABCA7 and ABCA1 [41,42,43,44].

### 3.2. ABCA7 Expression and Organ/Tissue Distribution

In spite of their structural homology, the transcription of *ABCA7* and *ABCA1* is differently regulated. Although *ABCA1* gene expression is upregulated through heterodimerization of the liver-X-receptor (LXR) and the retinoid-X-receptor (RXR) under conditions increasing cellular cholesterol accumulation [45,46], cellular cholesterol depletion has been shown to increase *ABCA7* mRNA levels [47]. The similar regulation pattern is also observed in β-hydroxy β-methylglutaryl-CoA (HMG-CoA) reductase, low density lipoprotein receptor (LDLR), and sterol-responsive/regulatory element binding proteins (SREBPs), indicating the contribution of sterol regulatory element to transcriptional regulation [48]. Indeed, reporter cell assays have revealed that *ABCA7* expression is positively regulated by sterol through the SREBP2 pathway, which is oppositely involved in *ABCA1* transcription [47].

ABCA7 expression is detected in a variety of tissues/organs, which include brain, lung, adrenal gland, kidney, spleen, thymus, lymph node, testis, keratinocytes, and pancreatic islets, as well as blood cells (i.e., macrophages, erythrocytes, and platelets) [39,44,49,50]. In human brain cell cultures, *ABCA7* mRNA is the most abundant in microglia compared to other cell types [51]. While in situ hybridization analysis has demonstrated that ABCA7 expression is higher in hippocampal neurons than other areas or cells in the mouse brain [51,52], single cell-type RNA-sequencing has detected *Abca7*, not only in neurons and microglia, but also in various cell types, including oligodendrocytes, endothelial cells, and astrocytes in the mouse cortex [53].

Interestingly, the *ABCA7* gene is expressed as two variants, full-length cDNA (type I) and the shorter splicing variant cDNA (type II), in a tissue-specific manner. In peripheral tissues, type I ABCA7 expression is predominantly detected in bone marrow, whilst the type II ABCA7 is mainly expressed in the spleen and trachea [54]. In vitro experiments have shown that cells expressing type II ABCA7 less efficiently mediate lipid efflux compared to type I [54]. Thus, although further studies are needed, it has been hypothesized that the two *ABCA7* splice variants might serve different functions depending on cell types, as well as organs/tissues.

### 3.3. ABCA7 and Lipid Metabolism

As predicted from the structure, the major function of ABCA7 is likely to regulate lipid metabolism. Serum levels of total cholesterol and high density lipoprotein (HDL) were lower in female ABCA7-knockout (KO) mice compared to wild-type mice under fasting conditions, while there was no significant difference in serum fatty acid levels [52]. Shotgun lipidomic analysis has also demonstrated that *Abca7* deficiency alters the phospholipid profile in the mouse brain [55]. In human embryonic kidney (HEK)-293 cells, apolipoprotein-mediated efflux of cellular phospholipids was facilitated by expressing human ABCA7 [41,44,56,57]. While cholesterol and phosphatidylcholine (PC) are the major lipids exported by ABCA1, liquid chromatography-tandem mass spectrometry analyses revealed that lysophosphatidyl choline (LPC) and PC are predominantly released from BHK cells by ABCA7 overexpression [58]. However, in conflict with those results regarding the overexpression of ABCA7, a report shows that ABCA7 deficiency does not influence cholesterol and phospholipid efflux in mouse primary macrophages [52]. Thus, future studies should refine the physiological function of ABCA7 as a lipid transporter by assessing the effects depending on ABCA7 expression levels, cell types, and acceptors.

In addition to lipid efflux, the forced expression of ABCA7 has been shown to increase the amounts of intracellular/cell surface ceramide and intracellular phosphatidylserine (PS) in HeLa cells, resulting in cell cycle arrest [59]. Moreover, ABCA7 deficiency causes the disruption of lipid rafts on the plasma membrane of thymocytes and antigen presenting cells in mice, which is likely to be associated with the compromised development and function of natural killer T cells [60]. Together, these findings suggest that ABCA7 plays a role in maintaining intracellular lipid metabolism, thereby regulating cellular homeostasis.

### 3.4. ABCA7 and Phagocytosis

In *Caenorhabditis elegans*, CED-7 is one of the major adhesion molecules mediating the engulfment of apoptotic cells during embryogenesis [61]. As the orthologue of *ced-7* in mammalians has been predicted to encode ABC transporters from sequence similarity [62], ABCA family members are likely involved in the regulation of phagocytosis. The ABCA7 protein also shares 24% sequence identity and 54% sequence similarity with the CED-7 protein [63]. Indeed, when ABCA7 was deleted in mouse embryonic fibroblast BALB/3T3 cells, their phagocytic activity to fluorescently-labeled latex beads was significantly decreased [47]. While the phagocytosis of fluorescent polystyrene microspheres was enhanced by apoA-I or apoA-II in mouse macrophage J774 cells, the effect was ablated by the knockdown of ABCA7, but not by ABCA1 [64,65]. Consistent with these results, an in vivo ink-engulfment assay has shown that *Abca7* deficient peritoneal macrophages possess an impaired phagocytosis ability compared to those from wild-type mice [64]. Additionally, apoA-I-mediated phagocytosis of *Staphylococcus aureus* was suppressed by ABCA7 knockdown in J774 cells, reproducing the results using artificial polystyrene beads [64]. Another study also revealed that the phagocytosis of apoptotic neutrophils is reduced in macrophages from ABCA7 heteroinsufficient mice compared to control mice, while FcR-mediated phagocytosis for viable neutrophils coated with anti-CD18 antibody is not affected [66]. Although further studies are required to determine the molecular mechanism underlying the link between ABCA7 and phagocytosis, ERK signaling is likely involved in the pathway. Whereas the phosphorylation of extracellular signal-regulated kinase (ERK) is an important process for the phagocytosis of dying cells in response to apoptotic cells or the complement protein C1q, the event is diminished in ABCA7-deficient macrophages [66]. Thus, these results indicate that ABCA7 critically regulate phagocytic function in macrophages, contributing to immune responses along with the host defense system [65]. Because microglia are the resident macrophages of the central nervous system [67], it has been hypothesized that ABCA7 also mediates phagocytic activity in microglia, which may be involved in AD pathogenesis.

## 4. ABCA7 and Alzheimer Disease-Related Phenotypes

### 4.1. ABCA7, Neurobehaviors, and Aβ Pathology in Mouse Models

While the roles of ABCA7 in lipid metabolism and macrophage-mediated phagocytosis have been actively studied, its function in the central nervous system has received relatively less attention. Nonetheless, a study has shown that ABCA7 deficiency causes slight, but significant, effects on neurobehaviors in young mice [68]. Male ABCA7-KO mice failed to develop significant short-term novel object recognition at the age of 20 weeks, whereas anxiety, short-term spatial memory, and fear-associated learning were not affected [68]. The cheeseboard task test found that female ABCA7-KO mice had impaired spatial reference memory compared to control mice [68]. Although the sex-dependent phenotypes should be further elucidated, ABCA7 likely plays a role in maintaining neuronal homeostasis rather than neurogenesis [69]. In an aged mouse cohort composed of male and female mice (20–22 months old), spatial memory was significantly impaired in ABCA7-KO mice compared to control mice when analyzed with the Morris Water Maze test [55]. Thus, aging may be a critical factor exacerbating the deleterious effect on cognition caused by ABCA7 loss-of-function.

Several groups, including us, have demonstrated the contribution of ABCA7 to Aβ pathology by crossing ABCA7-KO mice with the amyloid AD model J20 [70], TgCRND8 [71], or APP/PS1 mice [55]. In J20 mice, ABCA7 deficiency aggravates amyloid plaque burden at around 17 months of age accompanied with increased insoluble Aβ levels but not soluble Aβ [70]. TgCRND8 mice lacking ABCA7 showed a substantial increase in the density of both diffuse and dense plaques at an early stage as young as 10 weeks old, where insoluble Aβ levels significantly increased but soluble Aβ was reduced [71]. Consistent with these results, our findings have also demonstrated that ABCA7 deficiency exacerbates amyloid plaque burden and increases soluble/insoluble Aβ42 in APP/PS1 mice at seven months of age [55]. Thus, accumulating evidence indicates that ABCA7 deficiency facilitates brain Aβ deposition in mouse models. In the following sections, we will discuss how ABCA7 is involved in the mechanisms of Aβ clearance and production.

### 4.2. ABCA7 and Microglial Aβ Clearance

As gene network analyses have demonstrated that microglial expressing genes including *CR1*, *SPI1*, the *MS4As*, *TREM2*, *CD33*, and *INPP5D*, as well as *ABCA7*, are involved in AD [17], contributions of microglia to the disease pathogenesis have become increasingly focused. While activated microglia produces pro-inflammatory cytokines and reactive oxygen species (ROS) in AD brains, microglia plays a critical role in the cellular uptake and proteostasis of Aβ [72]. Microglia can phagocytize Aβ aggregates, whereas soluble Aβ is taken up through fluid phase micropinocytosis [73]. As discussed above, ABCA7 has been shown to mediate phagocytosis in macrophages. Indeed, the capacity for macrophages [70,74] and microglia [74] from ABCA7-KO mice to take up oligomeric Aβ was significantly reduced compared to wild-type mice. Consistent with those results from in vitro experiments, the elimination of Aβ oligomers in the hippocampus is likely diminished in ABCA7-KO mice [74]. Since the number of plaque-associated Iba1-positive microglia is not affected by ABCA7 deficiency in APP mouse models [55,70], ABCA7 may directly regulate the phagocytic pathways, rather than migration ability, in microglia. In addition, in vivo microdialysis did not detect any significant difference in Aβ clearance from the interstitial fluid between control and ABCA7-KO mice with an APP/PS1 background [55]. Thus, it is possible that ABCA7 predominantly mediates the phagocytosis of Aβ aggregates, but not soluble Aβ species. Since other brain cell types, including astrocytes, neurons, and cerebrovascular cells, also play a critical role in cellular Aβ uptake and subsequent degradation [75], future studies should address how ABCA7 in those cells participates in brain Aβ elimination.

### 4.3. ABCA7 and APP Processing

Aβ is proteolytically cleaved from APP by processing through β- and γ-secretases [76]. Of note, ABCA7, ABCA1, and ABCG1 are likely involved in the APP processing pathway [77,78]. When ABCG1 or ABCA1 were overexpressed in a CHO cell line expressing human APP, Aβ generation was significantly reduced, although the transient expression of ABCA2 did not affect the Aβ level [78]. ABCA7 overexpression has also been demonstrated to reduce levels of the secreted sAPPα, sAPPβ, and Aβ in CHO-APP cells without affecting the activities of α-, β-, and γ-secretases [77]. On the other hand, suppressing endogenous ABCA7 by siRNA has been shown to facilitate β-secretase cleavage, resulting in increased secretions of sAPPβ, Aβ40, and Aβ42 in HeLa cells [71]. Consistent with these results, ABCA7 knockdown accelerates the production of murine Aβ40 and Aβ42 accompanied with increased β-site amyloid precursor protein cleaving enzyme 1 (BACE1) expression in mouse primary neurons [55]. These findings have been confirmed in ABCA7-KO mice, where ABCA7 deficiency facilitates APP processing and increases Aβ levels in mouse brains with or without the human APP transgene [55,71].

While the endocytosis of APP into endosomes is likely an important step for APP processing and Aβ generation [79], ABCA7 deficit results in enhanced APP endocytosis in microglia, which is predicted to account for increased Aβ production [71]. However, whether ABCA7 can directly interact with APP or indirectly regulate APP trafficking through alternate APP-interacting proteins, and if similar phenotypes are detected in other brain cell types, remains unclear.

In addition, ABCA7 deficiency induces endoplasmic reticulum (ER) stress, represented by the activation of the PERK-eIF2α pathway [55]. Since the phosphorylation of eIF2α has been shown to increase BACE1 levels [80], accelerated APP processing and Aβ production caused by ABCA7 deficiency may be partially explained through the ER stress-related pathway. While SREBP2 is a transcription factor for *ABCA7* [47], SREBP2 levels are increased in brains from ABCA7-KO mice [55]. Interestingly, the activation of the SREBP2 pathway likely upregulates BACE1 expression [81], which also might be involved in the mechanisms of ABCA7 deficiency, resulting in enhanced Aβ generation. Therefore, although further studies are needed, increasing evidence suggests that ABCA7 plays a role in regulating APP processing likely through diverse mechanisms. Since the main function of ABCA7 may be linked to lipid metabolism, it is critical to determine if the accelerated APP cleavage induced through ABCA7 deficiency is mediated by an altered lipid profile. Indeed, increased levels of cellular cholesterol and phospholipids have been shown to regulate APP processing ranging/starting from the non-amyloidogenic α-secretase pathway to stimulation of the β- and γ-secretase pathways [82,83,84].

## 5. Summary and Perspective

**Figure 2:**
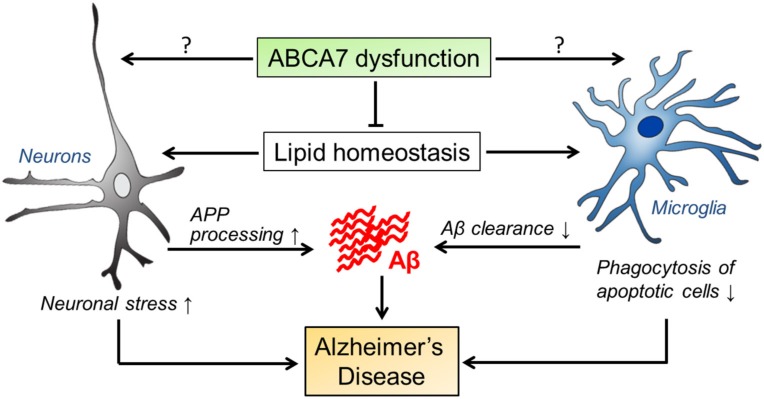
Possible pathogenic pathways mediated by ABCA7 in AD. ABCA7 dysregulation may influence the properties of brain cell types, in particular neurons and microglia, by disturbing brain lipid homeostasis and/or through unknown direct mechanisms. Those alterations likely facilitate APP processing and suppress cellular Aβ clearance, contributing to AD development. During the disease progression, ABCA7 deficiency may also exacerbate neuronal damages and diminish microglial phagocytic ability.

Since the discovery of *ABCA7* gene variants as susceptibility loci of AD from human genetics studies, a better understanding of the roles of ABCA7 in the central nervous system has been of high significance to explore the pathogenic pathways in AD. As PTC variants in *ABCA7* are associated with an increased risk for AD, subsequent studies have proven that ABCA7 deficiency exacerbates brain Aβ accumulation and AD-related phenotype using in vitro and in vivo models. Studies to date have mainly implicated two possible mechanisms whereby ABCA7 *loss-of-function* contributes to AD pathology; disturbing microglial Aβ clearance and accelerating APP processing. Furthermore, ABCA7 deficiency in microglia may compromise the elimination of diverse brain debris including apoptotic cells during AD progression. It is also possible that ABCA7 deficiency makes brain cells more vulnerable to Aβ toxicity and neuroinflammation in AD (Figure 2). Future studies should clarify the molecular mechanisms underlying the link between ABCA7 and the pathogenic pathways with a specific focus on the potential contribution from lipid metabolism. ABCA7 is likely expressed not only in neurons and microglia, but also in several other brain cell types. Thus, comprehensive single-cell type transcriptome analyses in human and mouse brains, and studies using conditional ABCA7 knockout mice may be necessary to determine cell-type specific contributions of ABCA7 to AD pathogenesis. It is also desired to determine if there is a common pathway between ABCA7-mediated pathways and those of other AD risk genes including *APOE4* and *TREM2*. These studies could provide us with novel insights to develop effective therapeutic strategies for AD. In addition, since lentivirus-mediated ABCA7 overexpression likely relieves the neurotoxicity of Aβ by promoting cell viability and reducing ER stress [85], the upregulation of ABCA7 through pharmacological approaches, including histone deacetylase inhibitors [86], may be beneficial in preventing and treating AD.

## Abbreviations

ABC	ATP binding cassette
ABCA7	ATP-binding cassette transporter A7
AD	Alzheimer’s disease
apoA	apolipoprotein A
apoE	apolipoprotein E
APP	amyloid precursor protein
Aβ	amyloid-β
BACE1	β-site amyloid precursor protein cleaving enzyme 1
ER	endoplasmic reticulum
ERK	extracellular signal-regulated kinase
GWAS	genome-wide association study
HMG-CoA	β-hydroxy β-methylglutaryl-CoA
HEK	human embryonic kidney
KO	knockout
LDLR	low density lipoprotein receptor
LPC	lysophosphatidyl choline
LXR	liver-X-receptor
MAF	minor allele frequency
MCI	mild cognitive impairment
NBD	nucleotide binding domain
OR	odds ratio
PC	phosphatidylcholine
PS	phosphatidylserine
PTC	premature termination codon
ROS	reactive oxygen species
RXR	retinoid-X-receptor
SNP	single nucleotide polymorphism
SREBP	sterol-responsive/regulatory element binding protein
